# Care of the Eyes

**Published:** 1880-09

**Authors:** 


### CARE OF THE EYES.

Do not read or write before sun up or after sun down. Let the light fall upon the page from behind.

Never read while lying down. Those whose eyes are weak should never read or sew by candle or gas light, nor by twilight. Suffer nothing to be applied to them unless by the special advice of an experienced physician. If the lids stick together in the morning on waking up, moisten them with the saliva, it softens and dissolves the matter sooner than any liquid known. The best and safest treatment for most affections of the eyes is rest, especially if weak or inflamed, rest from reading, writing or sewing, from every use of them which requires close observation, spending a large portion of the time out of doors, as then, large objects are mostly viewed. Persevere in this for weeks and months if necessary, and if not then relieved, consult a physician.

Avoid reading on horseback or in rail cars or any wheeled vehicle while in motion. Many persons will find that in reading before breakfast an effort is required to keep the sight clear but after breakfast, no such difficulty is experienced, the reason is, the eye under such circumstances is more or less inflamed, that is, has too much blood about it, but nature calls that excess of blood away to the stomach after eating, to enable it to perform its work more thoroughly. Therefore, persons with weak eyes should not read or write or do fine sewing on an empty stomach. Our Preceptor, Professor Dudley, who is among the very first of living Surgeons, used often to say, "Young gentlemen, never let anything touch the eye or ear stronger than luke-warm water." We have but one sight to lose, its preservation merits all our care, and it is unwise to tamper with, or experiment upon an organ so indispensable to our comfort, happiness and usefulness.

---

### TO PREVENT SLEEPING IN CHURCH.

Too many of our clergy have settled down into a conviction, that some how or other, their sermons in the aggregate, in the long run, will have a converting effect on the minds of their habitual hearers; and not being worked up into any expectation of an immediate conversion, their pulpit efforts, in the main, have degenerated into a tread-mill monotony; and it cannot but be expected that under such discourses, an active business man, who has been stirred up all the week by the merry jingle of dollars, can do otherwise than grow drowsy. He may be ashamed of it at the time; he may have conscience enough to lament it, and a will too, to fight against it, but the power, where is it? And any vain effort he may make to wake up, and get at the "*thread of the discourse*," only diverts his attention. For our part, we know of no available method of keeping wide-awake, within the sound of a dull sermon than this,

TAKE A NAP BEFORE YOU GO.

